# COMMENTARY ON “DOES AEROBIC EXERCISE AFFECT MEMORY, ATTENTION, WORKING MEMORY, AND FATIGUE AFTER ACQUIRED BRAIN INJURY? A SINGLE-BLINDED RANDOMIZED CONTROLLED PILOT STUDY”

**DOI:** 10.2340/jrm.v58.45973

**Published:** 2026-06-04

**Authors:** Palakdeep KAUR, Raju KUMAR, Nitin Kumar INDORA

**Affiliations:** Department of Physiotherapy, Maharishi Markandeshwar Institute of Physiotherapy and Rehabilitation, Maharishi Markandeshwar (Deemed to be University), Mullana, Ambala, Haryana, India, 133203.

*To the Editor,*

We read with great interest the pilot randomized controlled trial by Ingólfsdóttir et al. (1) examining the effects of aerobic exercise on cognitive function and fatigue after acquired brain injury. The authors are to be commended for addressing an important and clinically relevant aspect.

While reviewing the study, we would like to seek clarification on several methodological aspects that may influence interpretation of the findings.

The study had only 12 participants, even though a larger sample size was planned, and it was known to be underpowered. Small sample sizes are known to limit statistical power and increase the risk of type II error, which may explain why there were not many significant differences between groups in most outcomes (2, 3). We would like more information on how this limitation was taken into account when interpreting the negative results.

Second, various cognitive outcomes were evaluated; however, no correction for multiple comparisons was indicated. While methodologies for multiple testing are still being debated, some researchers argue that rigorous adjustments may not be necessary (4). Nonetheless, the interpretation of isolated statistically significant results – such as enhancement in working memory (PASAT) – should be undertaken with caution, especially when the majority of other outcomes did not reveal significant between-group differences. Elucidation on how the authors contextualize this finding within the overall pattern of results would be beneficial.

Third, including people who have had both a stroke and a traumatic brain injury makes the clinical picture less clear. Due to the unique pathophysiological mechanisms and recovery patterns linked to these conditions, variability in responses to exercise interventions has been documented in prior literature (2, 5). We seek further insight into how this diversity may have influenced the results and their interpretation.

Fourth, both the intervention and control groups underwent concurrent multidisciplinary rehabilitation. Given the observed improvements in both groups, it is essential to discern the independent effects of aerobic exercise from those of standard rehabilitation, particularly as structured rehabilitation programmes are recognized to enhance functional and cognitive outcomes (5).

Fifth, there was no record of activities performed post-exercise. As mental engagement following exercise may influence neuroplasticity and cognitive recovery, understanding how this variable could have altered the results would be beneficial (3).

In conclusion, although enhancements in executive processing speed were noted, the majority of primary outcomes did not exhibit significant inter-group differences. Prior research indicates that aerobic exercise may confer modest or domain-specific cognitive advantages (2, 3).

We sincerely appreciate the authors’ contribution to this field and believe that clarification of these points would further strengthen the interpretation and clinical relevance of the study findings.
