# Supplementary material for: COMMENTARY ON “DOES AEROBIC EXERCISE AFFECT MEMORY, ATTENTION, WORKING MEMORY, AND FATIGUE AFTER ACQUIRED BRAIN INJURY? A SINGLE-BLINDED RANDOMIZED CONTROLLED PILOT STUDY”
Source: J Rehabil Med. 2026 Jun 4;58:45973. doi: 10.2340/jrm.v58.45973 (PMC13244242; doi:10.2340/jrm.v58.45973)
Supplement: Related texts

# REPLY TO THE COMMENTARY ON “DOES AEROBIC EXERCISE AFFECT MEMORY, ATTENTION, WORKING MEMORY, AND FATIGUE AFTER ACQUIRED BRAIN INJURY? A SINGLE-BLINDED RANDOMIZED CONTROLLED PILOT STUDY”

**Authors:** Marika C. MÖLLER

**Affiliations:** From the Department of Rehabilitation Medicine, Danderyd University Hospital, Stockholm, Sweden. E-mail: marika.moller@regionstockholm.se

*To the Editor,*

Thank you for your thoughtful engagement with our article (1). We are pleased that the study has captured your interest, and we appreciate the opportunity to clarify and support accurate interpretation of our findings. We hope our responses will address the questions and welcome the opportunity to provide clarification and strengthen understanding of the study’s implications.

Beneath you will find our response to the questions point by point:

1. *Sample size and statistical power.* As noted, the study included fewer participants than originally planned. We fully acknowledge that the study was underpowered and that this limits the ability to detect between‑group differences. For this reason, the results should be interpreted as preliminary and exploratory. We found trends in differences before and after treatment in executive functions, which did not reach the significance threshold of 0.05 but could possibly have generated significant results in a larger study. Based on this, we believe that the results can encourage future studies of the effects of aerobic training on executive functions in more detail. The results could possibly have been clarified better by effect size estimates to inform future adequately powered trials.

2. *Multiple comparisons.* We agree that the absence of correction for multiple comparisons warrants caution in interpreting isolated significant findings. Given the exploratory nature of the pilot study, we chose not to apply formal adjustments, as this may increase the risk of type II error in small samples. Instead, we focused on reporting patterns across outcomes. The significant improvement in PASAT performance (*p* = 0.042) in the aerobic exercise group should therefore be viewed as a potential signal rather than a definitive effect on higher order cognitive functions. We explicitly refrained from drawing strong conclusions based on this single measure.

3. *Heterogeneity of stroke and traumatic brain injury.* Our patient group included individuals who had a stroke or a traumatic brain injury. Including both stroke and traumatic brain injury participants was a deliberate choice to reflect the clinical reality of mixed neurorehabilitation settings. We agree that these conditions differ in pathophysiology and recovery trajectories. On page 11, in the section discussing physiological responses in stroke vs TBI, we noted the following:

Stroke typically causes focal brain lesions, which can lead to asymmetric autonomic dysfunction, reduced baroreflex sensitivity, and therefore an attenuated heart rate response during exercise (6). In contrast, TBI more often results in diffuse autonomic dysregulation, characterized by sympathetic hyperactivity and reduced heart rate variability (7). These distinctions suggest that the 2 patient groups may respond differently to aerobic exercise, although we have not examined the extent to which this occurs.

Nonetheless, we acknowledge that variability may have influenced outcome patterns. In stroke patients, autonomic impairment may cause a slower-than-expected increase in heart rate for a given workload. As a result, they may need to exert more physical effort to keep their heart rate within 60–80% of their estimated maximum. Conversely, patients with TBI may show a faster-than-expected rise in heart rate relative to their actual level of exertion. Taken together, these patterns raise the question of whether heart rate is a reliable indicator of workload in individuals with TBI or stroke, thus making it more challenging to interpret the relationship between aerobic training and cognitive outcomes. Future studies with larger samples will be better positioned to examine diagnosis‑specific responses to aerobic exercise.

4. *Concurrent multidisciplinary rehabilitation.* This challenge was unavoidable in the clinical context and reflects real‑world practice, similar to what is described in Røe et al. (8). We agree that involving patient groups who are simultaneously engaged in multidisciplinary rehabilitation may have attenuated between‑group differences. As described in the Methods section on page 2, both the intervention group and the control group received multidisciplinary rehabilitation based on current guidelines and each patient’s individual needs. However, our intention was to evaluate whether aerobic exercise provides *additional* benefit beyond usual care.

This approach would be straightforward if both groups followed an identical, standardized rehabilitation programme – a “one‑size‑fits‑all” model. However, such an approach is incompatible with individualized rehabilitation, where treatment must be tailored to each patient’s specific needs. The pilot design does not allow us to isolate the independent contribution of aerobic exercise. This is an important consideration for future controlled trials. With a larger sample size, one might expect that differences in other treatment components would balance out across groups. In our study, however, the small number of participants makes it less likely that this balancing occurred.

5. *Lack of documentation of post‑exercise activities.* We appreciate the point raised regarding cognitive engagement following exercise. We did not systematically record post‑exercise activities, which is a limitation. We addressed this issue in part in the *Priming* section of the Discussion. If increased levels of BDNF and other neuroplasticity‑related factors are to meaningfully influence cognition following aerobic exercise, the aerobic training should presumably be followed by cognitive training to achieve optimal effects. However, we did not consider how long BDNF, and related neuroplasticity markers, remained elevated after the aerobic exercise. This “window of opportunity” is important, as cognitive gains are likely to be greatest when these levels are at their peak. With this in mind, one could reasonably expect greater improvements in cognitive recovery if patients engage in mentally demanding activities immediately after or during aerobic training as seen in recently published study by Wang et al. (9). In future studies it would be of interest to standardize post‑exercise cognitive load and investigate whether we might see more positive results regarding cognition if the patients were given cognitive demanding tasks right after the training, as this may interact with neuroplastic processes and influence cognitive outcomes.

Thank you for mentioning the potential positive effect of aerobic exercise on domain-specific cognitive advantages. We have in our article referred to Smith et.al (10) and the results of this meta-analytic review. As a pilot study, our work was designed to explore feasibility and generate hypotheses. However, larger, adequately powered studies with more homogeneous samples and tighter control of contextual variables are needed to clarify the cognitive effects of aerobic exercise in neurorehabilitation. We hope that our study, together with this exchange, contributes to the development of more rigorous future research in this important field.

## References

[1] Ingólfsdóttir E, Bergfeldt U, Berthold Lindstedt M, Bergfeldt L, Li TQ, Jonsson T, et al. Does aerobic exercise affect memory, attention, working memory, and fatigue after acquired brain injury? A single-blinded randomized controlled pilot study. J Rehabil Med 2026; 58: jrm44745. 10.2340/jrm.v58.4474541988967 PMC13213536

[2] Cumming TB, Tyedin K, Churilov L, Morris ME, Bernhardt J. The effect of physical activity on cognitive function after stroke: a systematic review. Int Psychogeriatr 2012; 24: 557–567. 10.1017/S104161021100198021996131

[3] Smith PJ, Blumenthal JA, Hoffman BM, Cooper H, Strauman TA, Welsh-Bohmer K, et al. Aerobic exercise and neurocognitive performance: a meta-analytic review of randomized controlled trials. Psychosom Med 2010; 72: 239–252. 10.1097/PSY.0b013e3181d1463320223924 PMC2897704

[4] Rothman KJ. No adjustments are needed for multiple comparisons. Epidemiology 1990; 1: 43–46. 10.1097/00001648-199001000-000102081237

[5] Billinger SA, Arena R, Bernhardt J, Eng JJ, Franklin BA, Johnson CM, et al. Physical activity and exercise recommendations for stroke survivors. Stroke 2014; 45: 2532–2553. 10.1161/STR.000000000000002224846875

[6] Gambassi BB, Almeida FdJF, Almeida AEAF, Ribeiro JA, Novais TMG, Schwingel PA, al. PASe. Effects of exercise training on cardiovascular and autonomic parameters in stroke survivors: a systematic review. Inter J Cardiovasc Sci 2019; 32: 408–413.

[7] Talbert LD, Kaelberer Z, Gleave E, Driggs A, Driggs AS, Baldwin SA, et al. A systematic review of the relationship between traumatic brain injury and disruptions in heart rate variability. Appl Psychophysiol Biofeedback 2024; 49: 523–540. 10.1007/s10484-024-09663-039222209

[8] Røe C, Bautz-Holter E, Andelic N, Søberg HL, Nugraha B, Gutenbrunner C, et al. Organization of rehabilitation services in randomized controlled trials: which factors influence functional outcome? A systematic review. Arch Rehabil Res Clin Transl 2022; 4: 100197. 10.1016/j.arrct.2022.10019735756983 PMC9214333

[9] Wang X, Zhang J, Wang X, Wang S, Zhang Y, Yang Y, et al. Multiscale mechanisms of exercise-induced neuroplasticity: from molecular pathways to network dynamics and behavioral adaptation. Brain Sci 2026; 16: 294. 10.3390/brainsci1603029441892637 PMC13025403

[10] Smith PJ, Blumenthal JA, Hoffman BM, Cooper H, Strauman TA, Welsh-Bohmer K, et al. Aerobic exercise and neurocognitive performance: a meta-analytic review of randomized controlled trials. Psychosom Med 2010; 72: 239–252 10.1097/PSY.0b013e3181d1463320223924 PMC2897704
